# Experimental Investigation of BFRP Tendons under Monotonic and Hysteretic Loadings

**DOI:** 10.3390/polym13213722

**Published:** 2021-10-28

**Authors:** Yongwei Wang, Zhen Zhou, Yang Wei, Dongping Zhu

**Affiliations:** 1 Key Laboratory of Concrete and Prestressed Concrete Structures of the Ministry of Education, Southeast University, Nanjing 210096, China; klxzwyw@seu.edu.cn; 2 College of Civil Engineering, Nanjing Forestry University, Nanjing 210037, China; wy78@njfu.edu.cn; 3 Department of Civil and Environmental Engineering, University of Connecticut, Storrs, CT 06269, USA; zhudongping@hotmail.com

**Keywords:** basalt fiber reinforced polymer (BFRP), stiffness, loading rate, hysteretic behavior, anchor colloidal deformation

## Abstract

Basalt fiber reinforced polymers (BFRPs) have been widely used in structures because of their high strength and light weight advantages. The previous research neglected the effect of the bonded anchor which led to overestimated performances of the BFRP tendons. In this study, experiments on the monotonic and hysteretic behaviors were conducted to analyze the mechanics of the BFRP tendons with bonded anchors. After considering the influences of the loading rates and initial prestressed displacements, the anchor colloidal deformations of the BFRP systems were measured and regressed. The results showed that the bearing and elongation capacities of the BFRP tendons, which exceed 3.3% and 140 kN, respectively, were independent of the loading rate and initial prestressed displacement under the monotonic and hysteretic loadings. The colloidal deformations, which accounted for about 20% of the whole deformation of the BFRP tendons, cannot be neglected in the predicted responses of the BFRP tendons. The regressed formulas of the anchor colloidal deformation could effectively anticipate the monotonic and hysteretic responses of the BFRP tendons. Therefore, the BFRP tendons with relatively stable mechanical properties are suitable for further practical application.

## 1. Introduction

For decades, concrete structures have undergone corrosion of steel bars which has led to premature failure and high repair costs. The traditional materials cannot meet the construction requirement of a long-span bridge or the convenience of rehabilitation. For self-centering structures that focus on controlling the residual drifts, the post-stressed strand system is essential to provide the restoring force [1,2]. However, the low elongation capability of the post-stressed strand limits the maximum deformation of a self-centering structure. Basalt fiber reinforced polymer (BFRP) tendon, a novel material with light weight, stable performance, and superior corrosion-resistant properties, has been developed as an alternative material to the traditional steel bar and post-stressed strand [3].

BFRP is an eco-friendly material as it is made from natural resources. It can resist impact load, high temperature, and chemical attack without easily fracturing [4,5]. Because of its orthotropic characteristic, the traditional anchorage system for steel strands is not suitable [6]. Considering the efficiency of practical application, a bonded-type anchor has been specially developed for the BFRP tendon [7,8]. Then, based on the experimental and numerical analyses, Chen [9] proposed an analytical model to estimate the mechanical behavior of the FRP joint with the bonded anchor. The prediction of the anchored BFRP system showed good accuracy with test results.

Recently, researchers have conducted experiments on the BFRP tendons and BFRP composites to investigate their mechanics. Through the fatigue experiment on the concrete beams with prestressed BFRP tendons, Atutis [10] observed that the mechanical property of the prestressed concrete beams had no noticeable degradation. Wang et al. [11,12,13] designed and investigated a long-span cable-stayed bridge with hybrid BFRP cables. The analysis results indicated that the seismic and economic performances of the proposed bridge were efficiently improved. Owing to its stable performance and elastic modulus, the BFRP tendon has been widely used to provide the self-centering force in a resilient structure. By combining the prestressed BFRP tendon and effective damper (bucking restrained brace or friction damper), novel braces were invented by Christopoulos et al. [14] and Zhou et al. [15,16], and desirable hysteretic performances of these braces were tested and verified.

Several earthquakes and experiments showed that the seismic response is a dynamic loading, not a static loading [17,18]. Even though the BFRP tendons present a stable property in engineering, current studies need to focus on the dynamic behaviors of the BFRP tendons in self-centering deceives. In previous research, the BFRP tendon with bonded anchors was regarded as an ideal elastic material, while the anchor colloid deformation of the BFRP tendon was ignored. However, unavoidable residual displacement was generated due to the colloid deformation, leading to the decreased prestressed force of the self-centering system. Therefore, the essential property and anchor behaviors of the BFRP tendon with bonded anchors are crucial.

It has been noted that many dynamic experiments on BFRP materials, like the sheet, laminate, and grid [19,20,21], have been carried out. Moreover, Wang et al. [22,23] investigated the fatigue behavior of the prestressed BFRP tendons with 6-mm diameter under low-stress range and investigated the good capabilities of the BFRP tendons. However, the behaviors of the BFRP tendons with greater diameters and their anchor performances under a high loading rate also need attention. Therefore, to fully develop the advantages of BFRP, especially for self-centering applications [16,24], the monotonic and hysteretic behaviors of the BFRP tendons should be tested and predicted, considering the anchor colloid deformation.

In the present study, a series of tests were conducted to investigate the monotonic and hysteretic behaviors of the BFRP tendons with bonded anchors. To fully evaluate the mechanical properties, the experiments considered the different parameters of the BFRP tendons, including the loading rates and the initial prestressed displacements. Then the force displacement and stiffness responses of the BFRP tendons during the entire loading process were obtained from the tests, considering the anchor colloidal deformations. Based on the regressed anchor colloidal deformations, the monotonic and hysteretic responses were calculated and compared with the test results.

## 2. Experiment Program

### 2.1. Specimen Description

**Figure 1:**
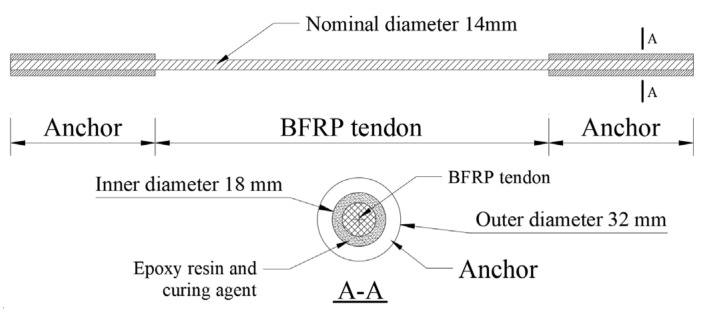
Configuration of the BFRP tendon with bonded anchors.

The nominal diameter of the test BFRP tendons was 14 mm with an area of 153 mm^2^. The configuration of the bonded anchors for the BFRP tendons is depicted in Figure 1. The anchors were fabricated by a circular steel tube, and the length of each anchor was 450 mm with the inner and outer diameters of 18 mm and 32 mm, respectively.

According to the test guidelines of the FRP tendon in ACI 440 [25], the length of the tested FRP tendons should not be less than 40 times the diameter of the tendon, which is calculated as 560 mm. Meanwhile, referring to the construction of a scaled self-centering brace with prestressed BFRP tendons in a building [15], the length of the tested BFRP tendons was 980 mm, and the length of the BFRP tendons with bonded anchors was 1880 mm.

A reliable anchor process was developed considering the significant influences of anchorage conditions on the BFRP tendon responses. Firstly, epoxy resin and curing agent were heated together until they were uniformly mixed. Secondly, the mixed colloid was poured into the anchor, and one end of the BFRP tendon was inserted into the anchor. Then, the anchor was cured for 30 min under a constant temperature of 120 °C. As a result, mixed colloids could effectively attach to the surface of the BFRP tendon. After the anchor was naturally cooled, it was placed in a warm environment for about 24 h. Then, the other end of the BFRP tendon was also anchored by repeating the same anchoring procedure. After the two ends were anchored, the BFRP tendon with bonded anchors was stored for 7 days before the experiment could be carried out.

### 2.2. Test Setup

**Figure 2:**
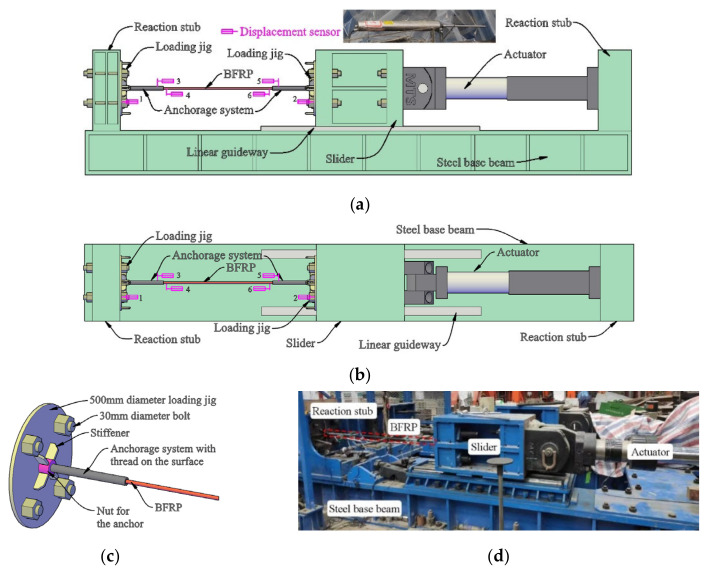
Setup for uniaxial loading of BFRP tendon: (**a**) side view; (**b**) top view; (**c**) fixing detail of BFRP; (**d**) experiment photo.

The experimental setup is shown in Figure 2. A 1000-kN actuator was placed along the axis of the specimens to impose the monotonic and hysteretic loadings along the axial of the BFRP tendons with bonded anchors. The two loading jigs on two sides of the specimen were bolted to the reaction stub and slider respectively by eight high-strength bolts with 800 N·m torque to prevent assembly deviation. The linear guideway fixed on the steel-based beam guaranteed the uniaxial displacement of the slider along the length direction of the specimen.

Several groups of anchors were fabricated using the circular steel tube to fix the BFRP tendons with bonded anchors. Then the thread was made on the outer surface of each anchor. One nut and several stiffeners were welded on the surface of each loading jig. The specimen was connected to the loading jigs at the two ends through the thread outside of the anchor, as shown in Figure 2c.

The displacement sensors used in this test were a kind of linear variable differential transformer (LVDT), as shown in Figure 2a. Through the data acquisition system, these sensors converted the pulse signals into displacement signals. Before the test, all displacement sensors were recalibrated to guarantee measurement accuracy. To measure the relative displacements between the jigs, slider, and reaction stub, displacement sensors 1 and 2 were placed near the two loading jigs. Results showed that the connections by high-strength bolts were slip-critical. The displacement results by displacement sensors 2 and the controlled system of the actuator also presented nearly the same values during the whole tests. Thus, displacement sensors without malfunction can work properly under high loading rates. Displacement sensors 3 and 5 were placed at the ends of anchors near the BFRP tendon, and displacement sensors 4 and 6 were placed at the ends of the BFRP tendons near the anchors. Therefore, the differences between the records from displacement sensors 3 and 4, and 5 and 6, correspond to the anchor colloid deformations at the two ends.

To avoid the influence of the high-frequency vibration generated by loading deceive, all the results measured by the displacement sensors were revised by a low-pass filter with a cut-off frequency of 20 Hz. Therefore, the influence of self-vibration of the displacement sensors under the high loading rate was avoided.

## 3. Testing Requirements and Validation

In a dynamic test with a high loading rate, it is crucial to verify the reliability of the test results through the stress equilibrium [26]. For the static or quasi-static test, an elastic wave has sufficient time to travel through the specimen several times. However, for the high loading rate test, an elastic wave must propagate inside the specimens for at least one cycle. Therefore, to meet the requirement of a test with high loading rates, the time for an elastic wave to travel a round trip in the BFRP tendon with bonded anchors can be calculated as follows:

(1) $$t=\frac{2L_{f}}{c}$$

where *L_f_* is the total length of the BFRP tendon system (1880 mm in this experiment); *c* is the elastic stress wave velocity in the BFRP tendon and can be expressed as follows:

(2) $$c=\sqrt{\frac{E}{\rho }}$$

where *E* and *ρ* are Young’s modulus and mass density of the material, respectively. For the BFRP tendon of 14-mm diameter, the *E* and *ρ* are 38 GPa and 2600 kg/m^3^, respectively [27,28]. The elastic stress wave velocity, *c*, is 3823 m/s. Therefore, it takes about 0.98 ms for the stress wave to propagate through the BFRP tendon. Under a loading rate of 200 mm/s, the BFRP tendon fractures at about 170 ms. Even under the maximum loading rate, the minimum loading time is 32.5 ms and sufficient for the elastic stress wave to propagate inside the specimens more than 30 times.

**Figure 3:**
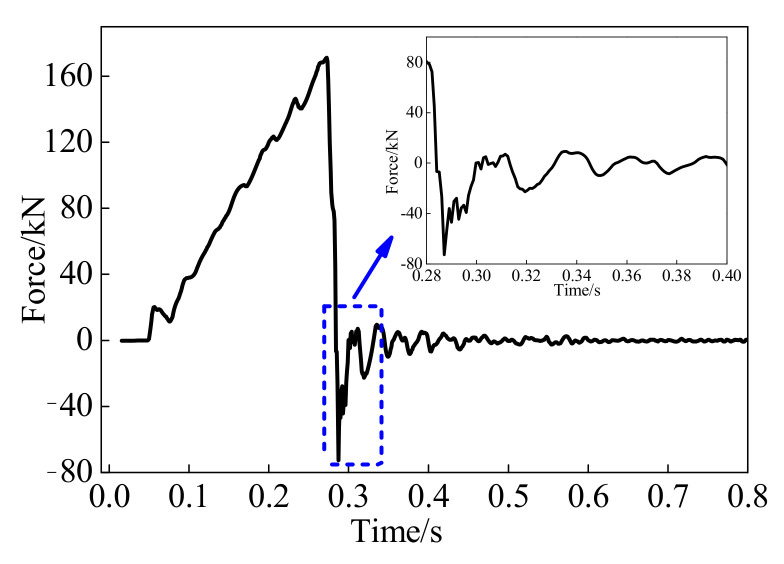
Free vibration of test system after a specimen fractured.

Moreover, the natural period of the whole test system, including the actuator, loading jags, slider, reaction stub, and BFRP tendon with bonded anchors, requires careful examination. If the natural period of the test system is longer than the loading time of the BFRP tendon, the load cell in the actuator may not correctly track the true applied force owing to the test system and specimen interaction. Through the free vibration of the whole test system, the estimated natural period is obtained and compared to the loading time. After that, the loading rates can be verified [26]. A typical force-time history after a specimen fractured is depicted in Figure 3. It can be estimated that the natural period of the whole test system is about 18 ms, which is shorter than the fracture time for a specimen of 170 ms, with a minimum loading time of 32.5 ms.

## 4. Monotonic Behavior

### 4.1. Failure Mode

A displacement-controlled loading protocol was adopted in this paper. Under the monotonic loading condition, the specimens were directly loaded to fracture under the loading rates of 0.1 mm/s, 50 mm/s, and 200 mm/s.

**Figure 4:**
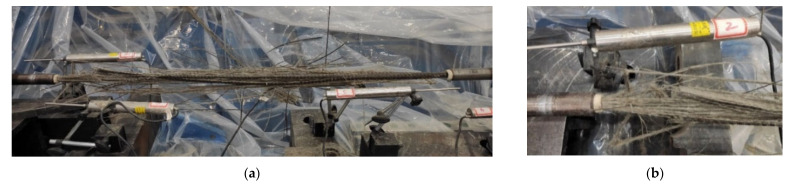
Fracture of the BFRP tendon: (**a**) failure mode; (**b**) anchor without obvious slip.

The fracture processes of all the BFRP specimens were similar. When the loading displacement was smaller than 27 mm, no apparent damage was observed in the BFRP tendon. Once the loading displacement reached approximately 27 mm, according to the 77% of the deformation capability, a brittle sound was emitted, and some surface BFRP fibers fractured. As the displacement increased, the fibers fractured from the outer surface to the inside until the BFRP tendons eventually failed. The measured force and displacement at this moment represented the bearing and deformation capacities, respectively. It can be observed that the fractured BFRP tendons were like a broom, while the anchors were still effectively anchored the BFRP tendons at the two ends without apparent anchorage slip (Figure 4).

### 4.2. Test Results

Table 1 lists the primary results of the BFRP tendons with bonded anchors under monotonic loading. The ultimate elongation of the BFRP tendons with bonded anchors considered the anchor colloidal deformations, while that of the BFRP tendon only calculated the deformation of the BFRP tendons by reducing the maximum colloidal deformations when the specimen was fractured.

The deformation capability of the BFRP with bonded anchors was about 35 mm, while the maximum colloidal deformation was greater than 7 mm. The colloidal deformation accounted for about 20% of the whole deformation. Therefore, the influence of colloidal deformation cannot be neglected.

**Figure 5:**
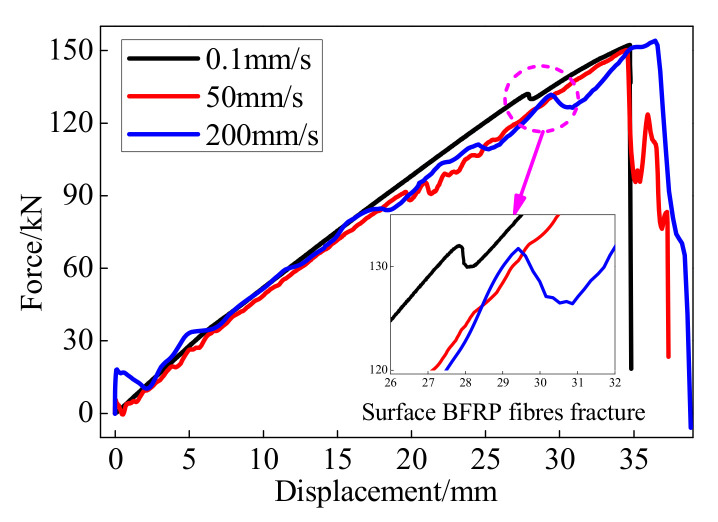
Monotonic performance of the BFRP tendons.

Figure 5 shows the monotonic displacement-force curves of the BFRP tendons under different loading rates. Since the BFRP tendon is a composite material made by a large amount of BFRP fibers, the property of BFRP tendons along the length direction is slightly different. The initial creak often generates at the weakest position on the BFRP surface. When the loading displacement was nearly 27 mm, a brittle sound was emitted and the surface fibers of the BFRP tendons began to fracture. Therefore, the axial force and the stiffness of the BFRP tendons abruptly decreased, as shown in Figure 5. Then the other fibers kept working until the progressive crack of the BFRP fibers led to the final failure of the BFRP tendons.

The BFRP tendons behaved approximately as linear elastic material until the points of fracture. The ultimate elongation of the BFRP tendons exceeded 2.7% with a slight difference, while that of the BFRP tendons with bonded anchors exceeded 3.5% owing to the anchor colloidal deformation. Although the force-displacement curves of the BFRP tendons under the loading rates of 50 and 200 mm/s had some fluctuations, the bearing and deformation capabilities were slightly influenced. Hence, the BFRP tendons exhibit stable monotonic performance. Moreover, when using the same anchor system to the BFRP tendons, the effect of anchor colloidal deformation on the monotonic behavior of the BFRP tendon cannot be neglected.

### 4.3. Anchor Colloidal Deformation

**Figure 6:**
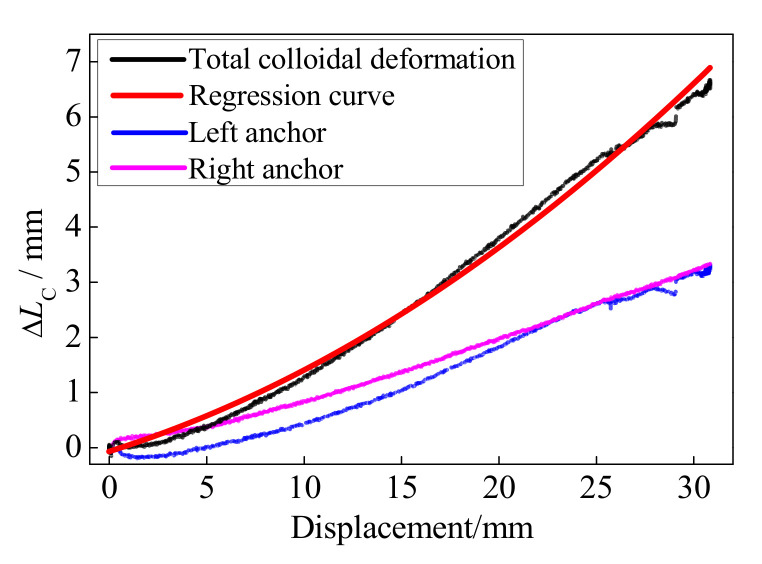
Anchor colloidal deformations.

Based on the records of the displacement sensors (Figure 2), the left and right anchor colloidal deformations are illustrated in Figure 6. With the increase in the loading displacement, the anchor colloidal deformations increased correspondingly. Owing to the anchor colloidal deformation during the test, the prestressed force loss for the BFRP tendon is unavoidable. Consequently, further research on the colloidal deformation is required to anticipate the force-displacement curve of the BFRP tendon. The previous research [29] showed that the stress-strain curve of the epoxy resin could be regarded as a linear relationship under small stress. After reaching the ultimate strength, the epoxy resin softens. The strain significantly increases, but the stress decreases. Because the epoxy resin is the main component to fix the anchors and BFRP tendon, its performance greatly influences the anchor colloidal deformation. Therefore, the linear relationship between the anchor colloidal deformation (strain) and loading force (stress) [28] is inaccurate.

Based on the experimental results of the anchor colloidal deformations, the relationship between the anchor colloidal deformation, Δ*L_C_*, and the loading displacement, *D_L_*, is obtained based on the regression formula.

(3) $$\Delta L_{C}=0.00376D_{L}{}^{2}+0.1097D_{L}$$

The units of Δ*L_C_* and *D_L_* are both mm. Figure 6 compares the experiment and regression results, depicting a good agreement. Based on Equation (3) and the basic property of the BFRP tendon, the force-displacement curve considering the anchor colloidal deformation can be calculated as follows:

(4) $$F_{BM}=k_{BFRP}\cdot (D_{L}-\Delta L_{C})=k_{BFRP}\cdot (0.8903D_{L}-0.00376D_{L}^{2})$$

where *k*_BFRP_ is the stiffness of the BFRP tendons and is 5.93 kN/mm according to the abovementioned results.

**Figure 7:**
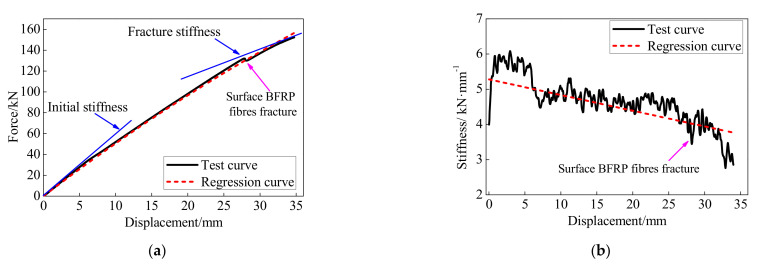
Comparison of the test and calculation results: (**a**) force-displacement curve; (**b**) stiffness.

The regressed and experimental responses of the BFRP tendons with bonded anchors are compared in Figure 7. The stiffness of the BFRP tendons in Figure 7b was calculated with the ratio of the incremental force and incremental deformation at each load step. The tangent stiffness and axial force of the BFRP tendons gradually decreased with greater loading displacement due to the colloidal deformation. Meanwhile, the surface BFRP fibers were gradually fractured during the test process, leading to a decrease in the force of the BFRP tendon. Therefore, as the test was controlled by displacement with the same incremental deformation at each loading step, the incremental force decreased with the increase in the loading displacement.

The regression model matches with the test curve and effectively simulates the stiffness degradation during the test process. Although the regression curve cannot predict the abrupt decrease in the loading displacement of 27.88 mm, the regression reveals the same tendency as the test results. Moreover, when the loading displacement was greater than 5.69 mm, corresponding to the 16% of the deformation capability, the stiffness of the BFRP tendons had an abrupt decrease. The main reason is that the epoxy resin softened after reaching its ultimate bearing capability. Then the test stiffness of the BFRP tendons gradually decreased and presented the same tendencies with the regression model. Therefore, the regression model considering the anchor colloidal deformation can effectively simulate the monotonic behavior of the BFRP tendons with bonded anchors.

## 5. Hysteretic Behavior

### 5.1. Loading Protocol

**Figure 8:**
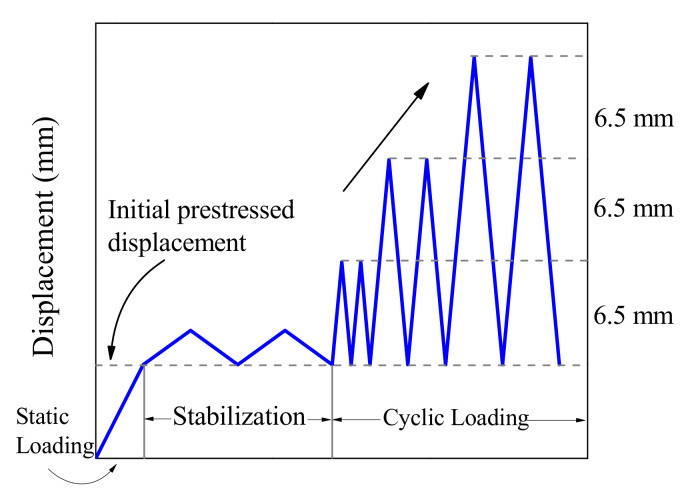
Cyclic protocol for the BFRP tendons with bonded anchors.

In the hysteretic loading condition, all specimens were initially prestressed under a loading rate of 0.1 mm/s before the cyclic loading. The initial prestressed displacements were selected as 4 mm (15% of the deformation capability), 8 mm (25% of the deformation capability), and 12 mm (35% of the deformation capability) to simulate the prestressed force in actual engineering. Then the specimen was applied with a slight loading displacement under a slow loading rate for stabilization. The loading protocol had an incremental displacement amplitude of Δ = 6.5 mm, representing the 1% story drift for the scaled prototype structure. Each loading amplitude was cycled twice to verify the stability of the BFRP tendon, as illustrated in Figure 8. After each loading amplitude, the actuator was returned to the initial prestressed position and prepared for the next loading.

Two different types of loading protocols in the hysteretic loading experiment, including the constant speed and constant frequency, were selected. For constant frequency loading, the loading frequency was set to 1.92 Hz regardless of the amplitude to analyze the behavior of the BFRP tendons under a gradually increased loading rate. The hysteretic loading sequences of the BFRP tendons are listed in Table 2.

### 5.2. Influence of the Loading Rate

Based on the loading sequences 1 to 7 and 14 listed in Table 2, the hysteretic experiments of the BFRP tendons were carried out under different loading rates. All specimens under hysteretic loading were fractured the same as those under the monotonic loading (Figure 4), and the phenomenon was not discussed. Therefore, this type of anchor applied in the specimen can efficiently anchor the Φ14-mm BFRP tendons.

**Figure 9:**
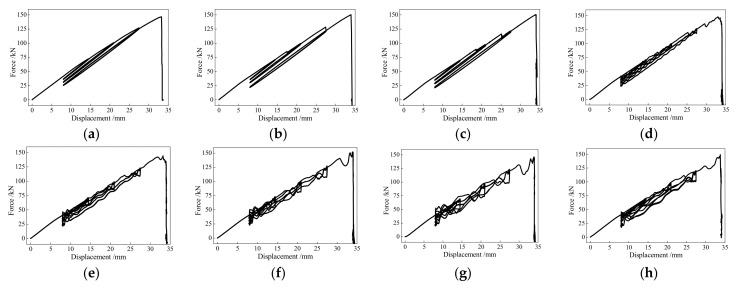
Hysteretic performance of the BFRP tendons under different loading rates: (**a**) 0.1 mm/s; (**b**) 1.0 mm/s; (**c**) 10 mm/s; (**d**) 50 mm/s; (**e**) 100 mm/s; (**f**) 150 mm/s; (**g**) 200 mm/s; (**h**) 1.92 Hz.

Figure 9 and Table 3 depict the hysteretic behaviors and basic results of the BFRP tendons under the different loading rates. With the increases in the loading displacement and the number of loading cycles, the prestressed forces of the BFRP tendons decreased after they returned to the initial prestressed displacement.

The BFRP tendons under hysteretic loading also exhibited stable behaviors, and the deformation and bearing capacities were slightly affected by the loading rate. For the eight specimens, the maximum differences of the elongation and bearing capacities only accounted for 2.4% and 5.2% of the ultimate deformation and bearing capacities, respectively. When the loading rate was greater than 100 mm/s, the stiffness of the BFRP tendons presented fluctuations, and this phenomenon was more obvious with the increase in the loading rate. However, the fluctuations had little influence on the fracture mode and ultimate performance, indicating the relatively stable responses of the BFRP tendons under the hysteretic loading.

**Figure 10:**
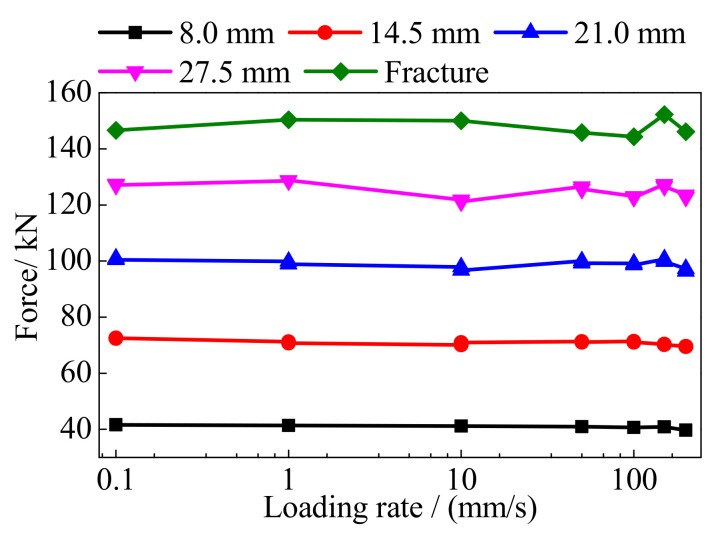
Peak force of the BFRP tendons.

Figure 10 shows the peak forces of the BFRP tendons under different loading rates and amplitudes. After excluding a few points with different tendencies, the BFRP tendons presented stable performances and insensitivity to the loading rate. The reason for the points with the different tendencies is that the BFRP tendons are made by a larger number of fibers with discrete performances. Their performances are inevitably affected by the processing technique, loading environment, and other factors.

The loss rate of the prestressed force, *γ*, is used to evaluate the residual prestressed force of the BFRP tendons after loading and can be expressed as follows:

(5) $$\gamma =\frac{F_{P}-F_{R}}{F_{P}}$$

where *F_P_* and *F_R_* are the prestressed force and the residual prestressed force after a loading amplitude, respectively.

**Figure 11:**
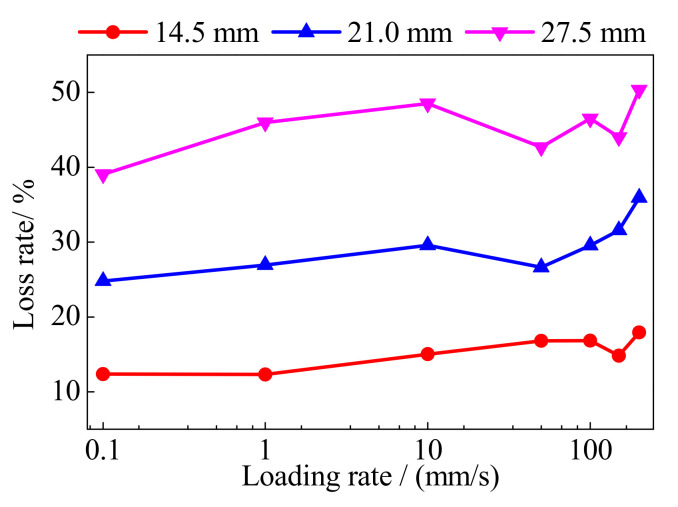
Loss rate of the prestressed force.

Figure 11 shows the loss rate of the prestressed force of the BFRP tendons under different loading rates. With the increase in the loading displacement, the loss rate of the prestressed force increased because of the greater anchor colloidal deformation. It is worth noting that the loss rate of the prestressed force increased with the greater loading rate. The maximum loss rate under the loading rate of 200 mm/s was greater than that under 0.1 mm/s, and the difference of prestressed force loss was greater than 3.65 kN which accounted for 11% of the prestressed force of the BFRP tendon. Owing to the composite material of the BFRP tendons, the bond behaviors of the BFRP fibers were affected by the high loading rate. However, 3.65 kN only accounted for less than 2.5% of the bearing capacity of the BFRP tendons. For the prestressed BFRP tendons, the deformation and bearing capacities require more attention. Thus, the increased loss of the prestressed force under a greater loading rate can be avoided by a greater prestressed force. Although the prestressed loss rates show slight loading rate dependency, they can be neglected when anticipating the hysteretic behavior of the BFRP tendons.

### 5.3. Influence of the Prestressed Displacement

**Figure 12:**
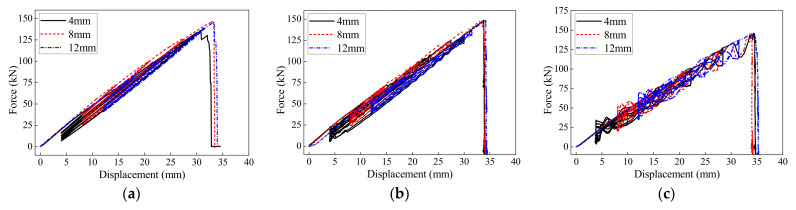
Hysteretic displacement-force curves of the BFRP tendons under different prestressed displacement: (**a**) 0.1 mm/s; (**b**) 50 mm/s; (**c**) 200 mm/s.

The loading sequences 1, 5, 7, and 8–13 were used to evaluate the influence of the initial prestressed displacement on the hysteretic behaviors of the BFRP tendons, and the results are shown in Figure 12. Under different loading rates and prestressed displacements, the BFRP tendons showed similar deformation and bearing capacities. The BFRP tendons were approximately fractured at the displacement of 34 mm and the force of 134 kN, except the one with a prestressed displacement of 4 mm under the loading rate of 0.1 mm/s. The slight difference was caused by the composite construction of the BFRP tendons, which is made by a large number of fibers with different behaviors. Although fluctuations were observed under high loading rates, the BFRP tendons still exhibited an obvious linear relationship between the force and loading displacement. Therefore, the BFRP tendons with stable monotonic and hysteretic behaviors are applicable in engineering practices.

### 5.4. Comparison of the Monotonic and Hysteretic Behaviors

**Figure 13:**
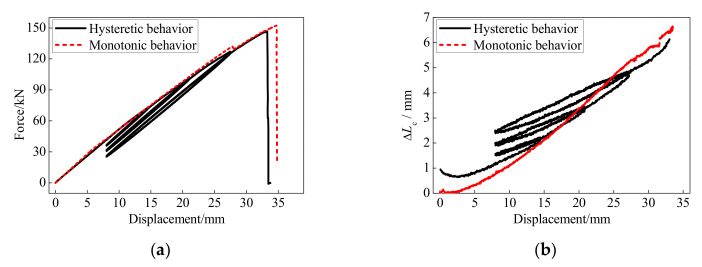
Comparison between the monotonic and hysteretic behaviors: (**a**) force-displacement curve; (**b**) anchor colloidal deformation.

The force-displacement and anchor colloidal deformation curves of the BFRP tendons under the monotonic and hysteretic loadings are illustrated in Figure 13. The BFRP tendons presented similar behaviors under the monotonic and hysteretic loadings. Although the deformation and bearing capacities of the BFRP tendons under the monotonic loading were 4% greater than that under the hysteretic loading, very similar skeleton curves are shown with hysteretic and monotonic behaviors. Therefore, the anchor colloidal deformations of the BFRP tendons under the monotonic loading can anticipate this under the hysteretic loading.

### 5.5. Secant Stiffness

**Figure 14:**
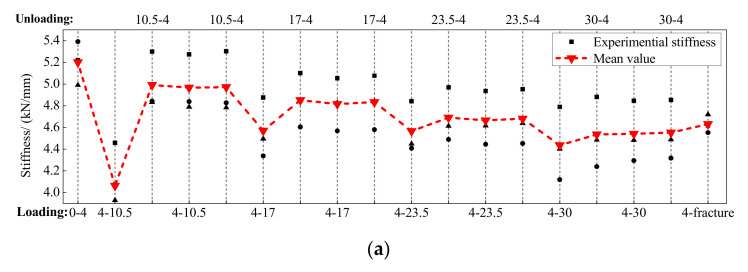

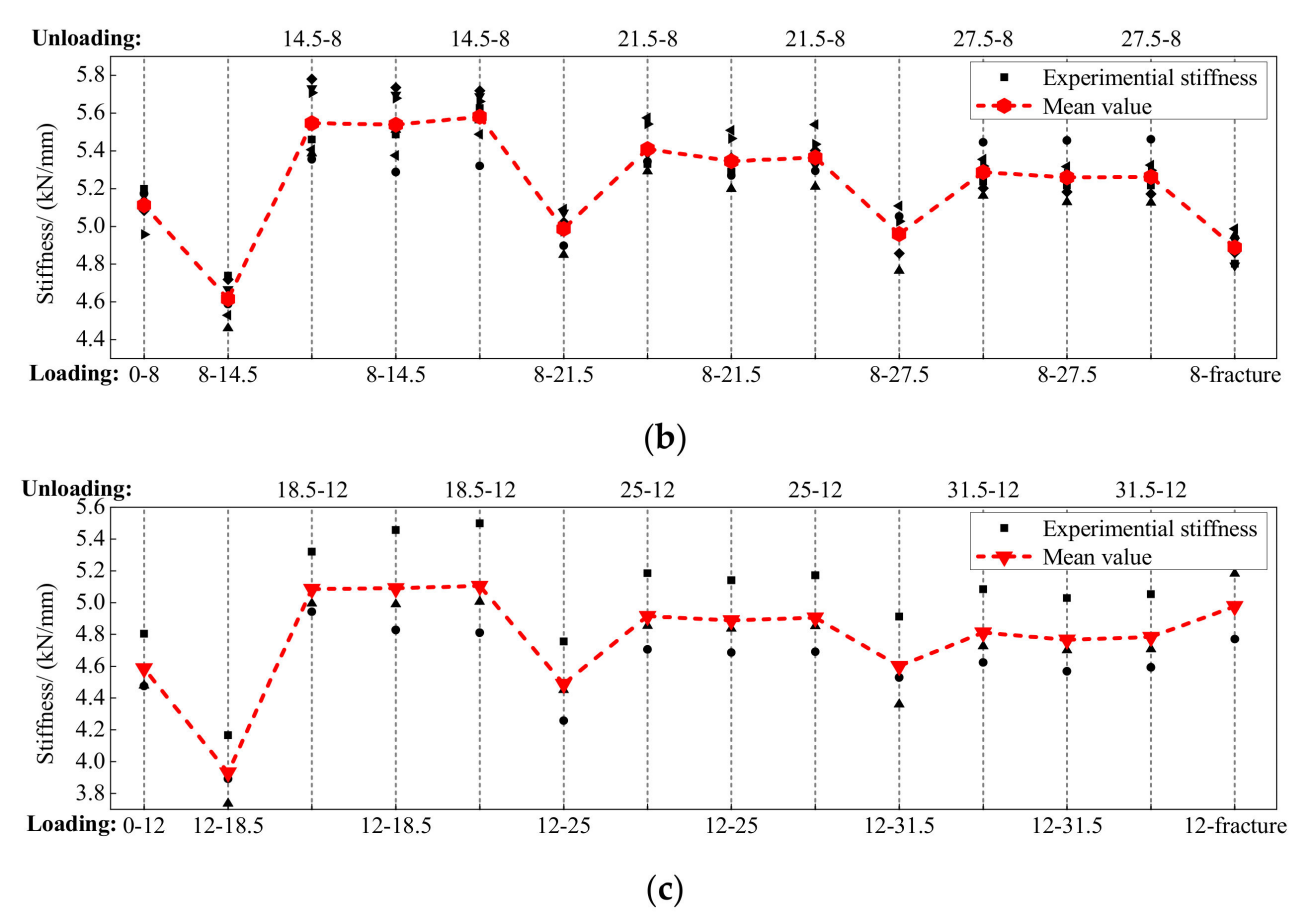
Secant stiffness of the BFRP tendons: (**a**) prestressed displacement of 4 mm; (**b**) prestressed displacement of 8 mm; (**c**) prestressed displacement of 12 mm.

The secant stiffness of the BFRP tendons during the whole loading process is calculated in Figure 14. The abscissa represents the entire loading process, and the abscissa values mean the start and end of the loading displacements. For example, the abscissa value of 4–10.5 means that the BFRP tendon was loaded from 4 mm to 10.5 mm, while the value of 10.5–4 implies that the BFRP tendon was unloaded from 10.5 mm to 4 mm. As mentioned above, each amplitude was conducted two times, and the second appearance of the abscissa value refers to the second loading and unloading cycle.

The secant stiffness of the BFRP tendons under the different loading rates presented similar tendencies. The BFRP tendons with different initial prestressed displacements depicted stable secant stiffness that was insensitive to the loading rate. When the BFRP tendons firstly reached a new amplitude, secant stiffness decreased compared to the previous stiffness. During the subsequent unloading and second cycle, the BFRP tendons presented stable and similar secant stiffness. The reason is that the anchor colloidal deformations elongated the length of the tested BFRP tendons with bonded anchors and reduced the prestressed forces in the first loading cycle. This phenomenon was more serious under the first loading amplitude, and the loading secant stiffness was the lowest during the whole loading process. Moreover, with the increase in the loading displacement, the secant stiffness gradually decreased because the BFRP fibers on the outer surface of the BFRP tendons were fractured, reducing the axial force and stiffness of the BFRP tendons.

### 5.6. Anchor Colloidal Deformation

**Figure 15:**
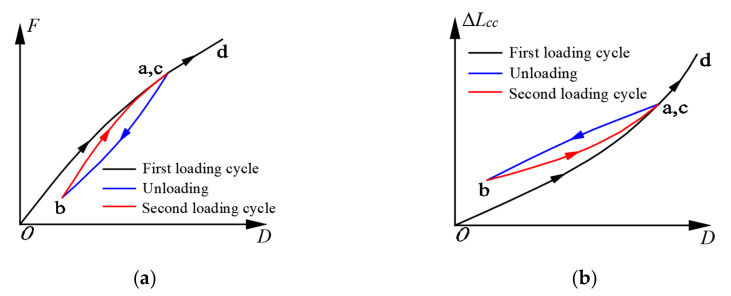
Conceptual diagrams of the BFRP tendons: (**a**) force-displacement curve; (**b**) anchor colloidal deformation.

As shown in Figure 13, the skeleton curve of the BFRP tendons under hysteretic loading can be predicted by the same regression formula of anchor colloidal deformation under monotonic loading. However, it is essential to evaluate the whole force-displacement curve of the BFRP tendons in the unloading and second cycle. Based on the hysteretic behavior of the BFRP tendons shown in Figure 12, the conceptual diagrams of the force-displacement and anchor colloidal deformation curves are illustrated in Figure 15.

In the loading stage of the first cycle (o-a), the force-displacement and anchor colloidal deformation curves of the BFRP tendons followed similar paths to those of the BFRP tendons under monotonic loading. Meanwhile, some inelastic anchor colloidal deformations were generated, and the prestressed forces of the BFRP tendons reduced correspondingly. In the first unloading stage (a-b), the BFRP tendons followed different paths than those in the initial loading stage (o-a). Therefore, the prestressed force at point b was smaller than the initial prestressed force due to the permanent anchor colloidal deformation. Although the BFRP tendons depicted a different force path in the loading stage of the second cycle (b-c) compared to the first cycle, the forces at point c were close to those at point a. In the second unloading cycle and the following loading stages, the BFRP tendons followed the paths of the first unloading and second loading cycles until they elongated to a new amplitude (point d).

Because of the different paths of the BFRP tendons in the first loading, unloading, and second loading cycles, the anchor colloidal deformations need to be analyzed separately. The anchor colloidal deformation in the initial loading cycle is similar to that under the monotonic loading (Figure 13), and the regression formula is the same as the BFRP tendons under the monotonic loading, as expressed in Equation (3). The regression formulas of the anchor colloidal deformations in the unloading cycle, $\Delta L_{C}^{U}$, and second loading cycle, $\Delta L_{C}^{S}$, are defined as follows:

(6) $$\Delta L_{C}^{U}=-0.00146D_{U}{}^{2}+0.0900D_{U}+0.0742$$

(7) $$\Delta L_{C}^{S}=0.00240D_{S}{}^{2}+0.0850D_{S}-0.1495$$

where *D_U_* and *D_S_* are the displacements in the unloading and second loading cycles, respectively.

**Figure 16:**
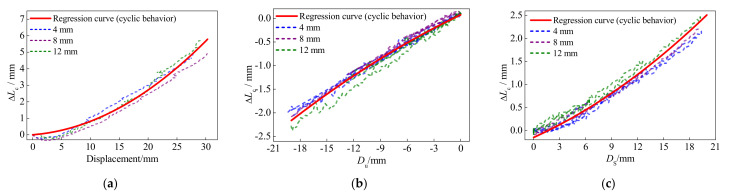
Anchor colloidal deformations of the BFRP tendons: (**a**) first loading cycle; (**b**) unloading cycle; (**c**) second loading cycle.

**Figure 17:**
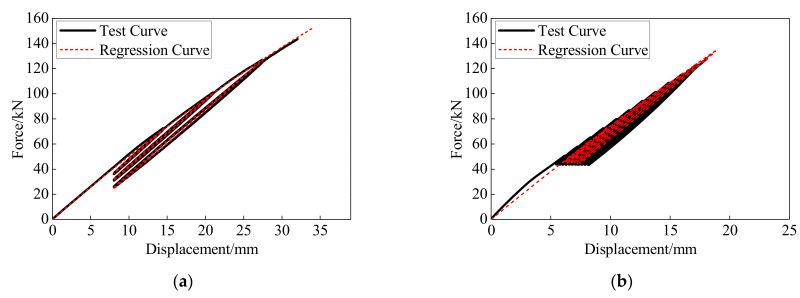
Comparison between the test results and calculation results: (**a**) results in this study; (**b**) result by Xie [28].

Figure 16 presents the comparison of the regression curves and anchor colloidal deformations in the experiment. The regression curves can accurately depict the variation trends of experimental anchor colloidal deformations. Based on Equations (6) and (7) and the basic property of the BFRP tendons, the comparisons between the calculation results and experimental results in this study and conducted by Xie [28] are shown in Figure 17.

The regression model for the hysteretic behavior of the BFRP tendons matches with the test curves and effectively simulates the different paths in the first loading, unloading, and second loading cycles. Thus, the regression model considering the anchor colloidal deformation can effectively simulate the hysteretic behavior of the BFRP tendons.

## 6. Conclusions

This study tested and analyzed the monotonic and hysteretic behaviors of the 18-mm diameter BFRP tendons with bonded anchors. The anchor colloidal deformations leading to the force and stiffness degradations were regressed. Based on the regression equation, the calculated monotonic and hysteretic responses were compared with the experimental results. The main findings are summarized below:

1. The deformation and bearing capacities of the BFRP tendons are slightly influenced by the loading rate and initial prestressed displacement under the monotonic and hysteretic loadings, and they exceed 3.3% and 140 kN, respectively. The stiffness and prestressed force loss of the BFRP tendons show some fluctuations with the increase in the loading rate.
2. The influence of the anchor colloid deformation cannot be neglected. The colloidal deformation accounts for about 20% of the deformation of the BFRP tendons and leads to the decreases in the axial force and stiffness of the BFRP tendons.
3. The hysteretic performances of the BFRP tendons present similar secant stiffness, but different force-displacement paths in the first loading, unloading, and second loading cycles.
4. Based on the regression formulas of anchor colloid deformations, the monotonic and hysteretic curves of the BFRP tendons were calculated and compared with the experimental results, presenting good agreements. Consequently, the BFRP tendons are suitable for further engineering because of stable and easily anticipated behaviors under the monotonic and hysteretic loadings.

## Figures and Tables

**Table 1:** Summary of the monotonic behavior of the BFRP tendons.

Loading Rate/(mm/s)	Failure Form	Bearing Capacity/kN	Deformation Capability/mm	Maximum Colloidal Deformation/mm	Ultimate Elongation/%	
					BFRP Tendons with Bonded Anchors	BFRP Tendons
0.1	Fracture	152	34.72	7.19	3.54	2.81
50	Fracture	150	34.65	7.26	3.54	2.79
200	Fracture	154	36.44	7.11	3.72	2.99
Mean	-	152	35.27	7.19	3.60	2.86

**Table 2:** Loading rates and frequency for hysteretic test.

Loading Sequence	Loading Type	Initial Prestressed Displacement/mm	Loading Rate/(mm/s)	Minimum Loading Frequency/Hz	Maximum Loading Frequency/Hz
1	Constant speed	8	0.1	0.000962	0.003846
2	Constant speed	8	1	0.00962	0.03846
3	Constant speed	8	10	0.0962	0.3846
4	Constant speed	8	50	0.48	1.92
5	Constant speed	8	100	0.96	3.84
6	Constant speed	8	150	1.44	5.76
7	Constant speed	8	200	1.92	7.69
8	Constant speed	4	0.1	0.000962	0.003846
9	Constant speed	4	50	0.48	1.92
10	Constant speed	4	200	1.92	7.69
11	Constant speed	12	0.1	0.000962	0.003846
12	Constant speed	12	50	0.48	1.92
13	Constant speed	12	200	1.92	7.69
Loading protocol	Loading type	Initial prestressed displacement/mm	Loading frequency/Hz	Minimum loading speed/(m/s)	Maximum loading speed/(m/s)
14	Constant frequency	8	1.92	50	200

**Table 3:** Summary of the hysteretic behaviors of the BFRP tendons.

Loading Rate	0.1 mm/s	1 mm/s	10 mm/s	50 mm/s	100 mm/s	150 mm/s	200 mm/s	1.92 Hz
Deformation capability/mm	33.26	33.93	34.02	33.53	33.20	33.95	33.92	33.76
Ultimate elongation/%	3.39	3.46	3.47	3.42	3.39	3.46	3.46	3.44
Bearing capacity/kN	146.64	150.37	150.02	145.81	144.34	152.23	146.11	149.25

## Data Availability

The data presented in this study are available on request.
